# Sex Differences in Epicardial Adipose Tissue: Association With Atrial Fibrillation Ablation Outcomes

**DOI:** 10.3389/fcvm.2022.905351

**Published:** 2022-06-13

**Authors:** Jing Zhu, Kaimin Zhuo, Bo Zhang, Zhen Xie, Wenjia Li

**Affiliations:** ^1^Department of Radiology, The First Affiliated Hospital of Chengdu Medical College, Chengdu, China; ^2^Department of Cardiology, The First Affiliated Hospital of Chengdu Medical College, Chengdu, China

**Keywords:** atrial fibrillation, major adverse cardiovascular events, epicardial adipose tissue, recurrence, sex

## Abstract

**Background:**

There are significant differences in the prevalence and prognosis of atrial fibrillation (AF) between sexes. Epicardial adipose tissue (EAT) has been found as a risk factor for AF. This study aimed to evaluate whether sex-based EAT differences were correlated with AF recurrence and major adverse cardiovascular events (MACE).

**Methods:**

In this study, postmenopausal women and age, BMI, and type of AF matched men who had received first catheter ablation were included. EAT volume was quantified based on the pre-ablation cardiac computed tomography (CT) images. Clinical, CT, and echocardiographic variables were compared by sex groups. The predictors of AF recurrence and MACE were determined through Cox proportional hazards regression.

**Results:**

Women were found with significantly lower total EAT volumes (*P* < 0.001) but higher periatrial/total (P/T) EAT ratios (*P* = 0.009). The median follow-up duration was 444.5 days. As revealed by the result of the Kaplan-Meier survival analysis, the women were found to have a significantly higher prevalence of AF recurrence (log rank, *P* = 0.011) but comparable MACE (log rank, *P* = 0.507) than men. Multivariate analysis demonstrated that female gender (HR: 1.88 [95% CI: 1.03, 4.15], *P* = 0.032), persistent AF (HR: 2.46 [95% CI: 1.19, 5.05], *P* = 0.015), left atrial (LA) dimension (HR: 1.47 [95% CI: 1.02, 2.13], *P* = 0.041), and P/T EAT ratio (HR: 1.73 [95% CI: 1.12, 2.67], *P* = 0.013) were found as the independent predictors of AF recurrence. Sex-based subgroup multivariable analysis showed that the P/T EAT ratio was an independent predictor of AF recurrence in both men (HR: 1.13 [95% CI: 1.01, 1.46], *P* = 0.047) and women (HR: 1.37 [95% CI: 1.11, 1.67], *P* = 0.028). While age (HR: 1.81 [95% CI: 1.18, 2.77], *P* = 0.007), BMI (HR: 1.44 [95% CI: 1.02, 2.03], *P* = 0.038), and periatrial EAT volume (HR: 1.31 [95% CI: 1.01, 1.91], *P* = 0.046) were found to be independent of MACE.

**Conclusion:**

Women had a higher P/T EAT ratio and AF post-ablation recurrence but similar MACE as compared with men. Female gender and P/T EAT ratio were found to be independent predictors of AF recurrence, whereas age and periatrial EAT volume were found to be independent predictors of MACE.

## Introduction

Atrial fibrillation (AF) has been found as the most prevalent cardiac arrhythmia in clinical practice, and it is correlated with an elevated risk of stroke and heart failure (1, 2). There are significant differences in the prevalence and prognosis of AF between the sexes, which are similar to other cardiovascular diseases (3). The risk of developing AF in women was 1.5–2.0 times higher than that in men (4). However, women with AF will have more severe symptoms and a poorer quality of life in contrast to men (5, 6). Several reports found that women had a higher risk of arrhythmia recurrence and periprocedural complications and hospitalization than men with AF (7–9). Although the results regarding the effect of sex differences on AF ablation outcomes have been controversial, an existing systematic review and meta-analysis proved that women were reported with lower AF-free survival than men (9, 10). However, the potential mechanisms of the observed sex difference in clinical outcomes following the AF post-ablation remain unclear.

Over the past few years, epicardial adipose tissue (EAT) has aroused increasing attention as it has been reported to play a role in the development and maintenance of AF and has a stronger relationship to AF presence and severity compared with other adiposity markers [e.g., waist circumference, waist/hip ratio, and body mass index (BMI)] (11–13). EAT, as the metabolically active visceral adipose depot closest to the myocardium, exhibits cell-to-cell contact or infiltration and shares the same blood supply as the myocardium, thus contributing to crosstalk with cardiomyocytes through direct paracrine and vasocrine pathways (11, 14). Some existing studies have suggested that when compared with total EAT, left atrial EAT is more significantly correlated with markers of atrial fibrillation (e.g., atrial structural remodeling and vulnerability), likely because the secretion of pro-inflammatory and anti-inflammatory adipokines by EAT around the left atrium could directly affect the left atrial wall and interact with cardiac autonomic nervous system (15–17). Thus, this study aimed to evaluate the sex-related difference in total EAT and periatrial EAT volume measured using cardiac computed tomography (CT) and correlate these with post-ablation AF recurrence and major adverse cardiovascular events (MACE).

## Methods

### Patient Population

In this study, all consecutive patients suffering from symptomatic AF who had received preprocedural cardiac CT for its ability to comprehensively evaluate pulmonary veins and left atrium, identify small variant anatomy, and rule out any suspicion of cardiac thrombus (18), and echocardiography examinations before the first-time catheter ablation between January 2018 and June 2021 at The First Affiliated Hospital of Chengdu Medical College were retrospectively recruited. The postmenopausal women and men matched for age, BMI, and type of AF were included. Clinical data were abstracted from the patient reports and the electronic medical records (e.g., demographic information, smoking, diabetes mellitus, hypertension, dyslipidemia, heart failure, coronary artery disease, history of stroke/transient ischemic attack (TIA), prior myocardial infarction, AF type (paroxysmal, persistent), and medications). The CHA_2_DS_2_VASC score was determined in accordance with the variables including congestive heart failure (1 point); hypertension (1 point); age 65–74 years (1 point); age ≥ 75 years (2 points); diabetes mellitus (1 point); prior stroke, TIA, or thromboembolism (2 points); vascular disease (1 point); and gender (female: 1 point) (19). Echocardiographic database was used to collect data on left ventricular ejection fraction (LVEF), left atrial (LA) dimension, and E/A-ratio. This study followed the principles in the Declaration of Helsinki and gained approval from the Institutional Review Board of our hospital.

### Cardiac Computed Tomography Imaging

All patients were scanned with a 128-row spiral CT system (SOMATOM Definition; Siemens AS). An intravenous injection protocol with 80 ml of 370 mg I/ml iopamidol (Shanghai Bracco Sine Pharmaceutical Corporation Ltd, China) was employed at an infusion rate of 5 ml/s. Subsequently, 40 ml saline was injected at the same flow rate. The contrast-enhanced cardiac CT scans were performed based on a retrospectively gated electrocardiogram (ECG)-triggered sequential protocol. To be specific, the scanning parameters included tube voltage, 120 kV; automatic tube current modulation, 100–400 mAs; thread pitch, 0.18; field of view (FOV), 145 mm × 145 mm; and rotation time, 0.3 s. Images were reconstructed (slice thickness, 0.75 mm and slice interval, 0 mm) using retrospective ECG gating (70%−80% R–R interval). The effective radiation ranged from 5 to 5.5 mSv.

### Left Atrial Volume Assessment

Left atrial volume (LAV) was calculated on the workstation (syngo MMWP OT 46806, VE40A; Siemens Medical Systems). The endocardial contour of the LA was manually outlined on each axial image slice. Every image was carefully ruled out because of the LA appendage and pulmonary veins. All voxels at each slice were added to derive LAV.

### EAT Volume Analysis

**Figure 1:**
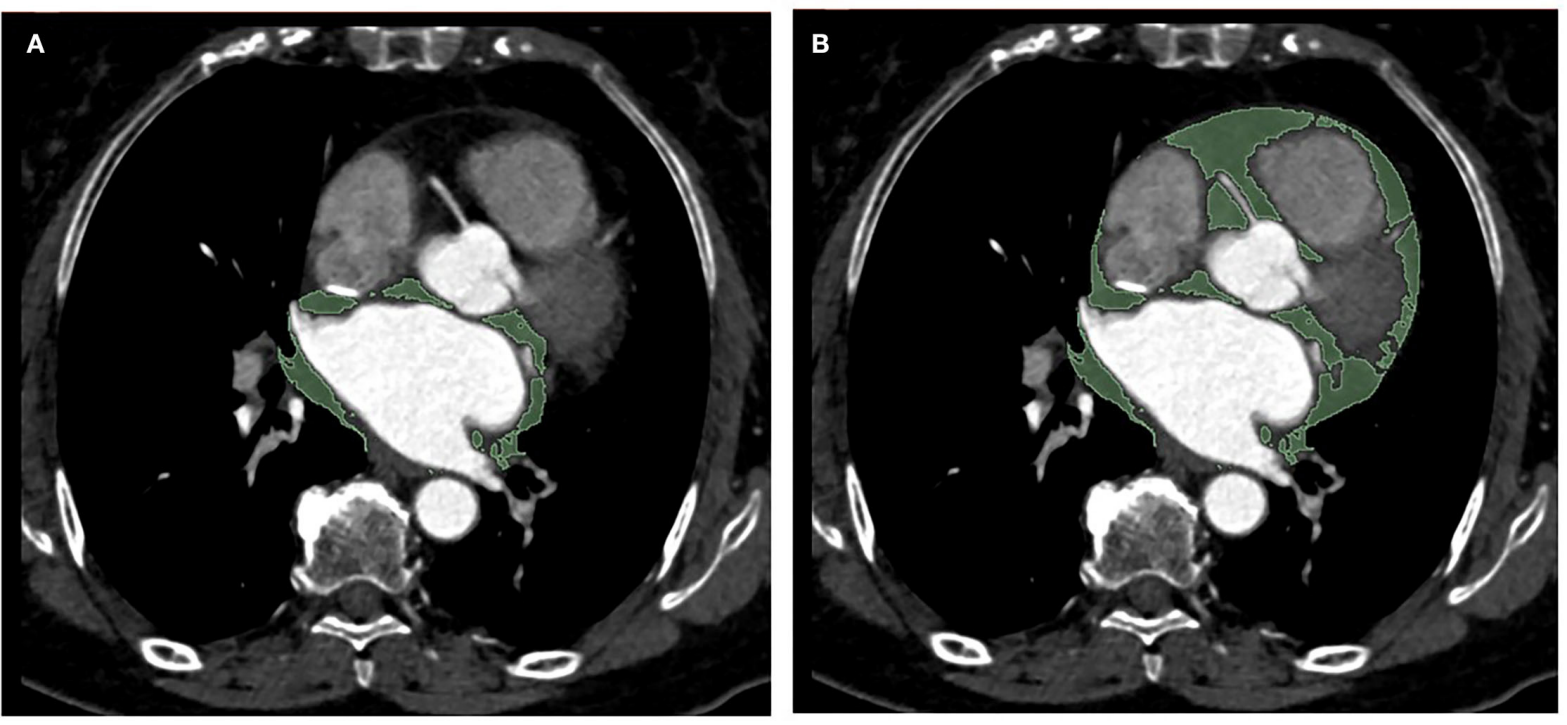
Representative example of periatrial EAT **(A)** and total EAT **(B)** measurement in a single axial CT image in a patient (area in green depict traced EAT). EAT, epicardial adipose tissue.

Epicardial adipose tissue volume measurement was performed with a 3D Slicer software (Boston, USA, 4.11.2 version). EAT has been defined as the adipose tissue between the pericardial visceral layer and the surface of the myocardium. The volumes of total EAT and periatrial EAT surrounding the left atrium were determined by manually tracing from the first section to the last section containing any images of the whole heart and the left atrium, respectively. The region outside the pericardium was excluded. EAT was automatically recognized using the software as tissue with HU between −195 and −15 for contrast-enhanced images as described previously (20). Subsequently, the region of interest (ROI) was examined and reviewed by the experienced operator (with 4 years of experience in cardiovascular imaging), and the voxels in the respective slice were summed to determine the total EAT volume and periatrial EAT volume (Figure 1). Moreover, the periatrial to total EAT ratios (P/T EAT ratio) of all patients were calculated. For the interobserver analysis, 50 individuals were randomly selected and then analyzed independently by two experienced radiologists (with 4 years of experience in cardiovascular imaging) blinded to clinical characteristics and CT findings. In terms of the intra-observer analysis, the results of the same 50 subjects above were measured again by one of the radiologists 2 weeks later.

### Atrial Fibrillation Ablation

All patients included in this study received catheter ablation using radiofrequency ablation, and the indications for the procedures were based on the current guidelines (21). A femoral vein puncture was performed to place the arterial sheath. Subsequently, electrodes were delivered into the apical and coronary sinus through the arterial sheaths for intracardiac electrophysiological examination to determine the location of the trigger of AF. The atrial septal puncture was performed to push heparin. Next, pulmonary vein and LA angiography were performed. A three-dimensional LA model was built by feeding a star-shaped magnetoelectric dual-positioning calibration catheter along the arterial sheath. Afterward, a cold saline pressure ablation adjustable elbow catheter (D133604IL, QiangSheng, China) was placed for circumferential pulmonary vein isolation; on that basis, a bidirectional block was achieved in the pulmonary vein based on Carto 3D electrical labeling. The ablation target was found as the earliest point of cardiac excitation at the onset of AF, and the endpoint was the disappearance of AF after ablation and its inability to be reevoked. Finally, the electrode was withdrawn, and the arterial sheath was removed when the sinus heart rate persisted for more than 15 min.

### Follow-Up Visits

All patients were followed after their first ablation procedures were completed. The follow-up examinations for AF monitoring were generally performed at 3 or earlier, 6, 9, and 12 months, then once or twice a year thereafter. The AF recurrence was determined in accordance with the clinical evaluation, 12 lead ECG, as well as 24-h Holter monitoring. One of the endpoints of recurrence was defined as AF episodes >30 s in duration documented on standard ECG recordings, event-activated ECG recordings, or 24-h Holter recordings.

Another endpoint of MACE comprised stroke/TIA, myocardial infarction, heart failure, major bleeding, cardiovascular hospitalization, and cardiovascular death during follow-up.

### Statistical Analysis

Continuous variables were expressed as mean ± standard deviation (SD) if normally distributed or median and interquartile range (IQR) if not normally distributed. Categorical variables were expressed as numbers (n) and percentages (%). Continuous variables were compared through the Student's *t*-test or Mann–Whitney U tests as appropriate, while categorical variables were compared through chi-square tests or Fisher exact tests as appropriate. The correlation of BMI with total EAT volume, periatrial EAT volume, and P/T EAT ratio was performed using Pearson correlation analysis. Intraclass correlation coefficients (ICC) were determined to evaluate interobserver and intraobserver reproducibility. The survival curve of AF recurrence and MACE was studied using the Kaplan–Meier method, and the comparison between sex groups was evaluated based on log-rank tests. The predictors of AF recurrence and MACE were determined using the univariable and multivariable Cox proportional hazard regression analysis. Variables with *P* < 0.1 were included in the multivariate Cox models. Receiver-operating characteristic (ROC) curve analysis was performed to evaluate the diagnostic performance and the optimal cutoff values of the P/T EAT ratio for AF recurrence. A two-tailed *P* < 0.05 was considered statistically significant. Statistical analyses were conducted using SPSS (version 22, IBM, Chicago, IL, USA), and graphs were generated using the Prism software (version 8, GraphPad, San Diego, CA, USA).

## Results

In this study, 71 men and 87 women were generally included. Table 1 and Supplementary Material 1 list the baseline characteristics of the study. The mean age was 69 ± 10 years, and 67 (42%) had persistent AF. There was no significant difference between men and women in age, BMI, hypertension, dyslipidemia, CAD, heart failure, prior myocardial infarction, stroke/TIA, and medications. Women had a lower proportion of smoking habits and a higher proportion of diabetics than men. Female patients had higher CHA_2_DS_2_VASC scores than male patients.

**Table 1:** Baseline characteristics of the study populations.

	**Male (*n* = 71)**	**Female (*n* = 87)**	**P-value**
Age, y	67 ± 11	70 ± 9	0.064
BMI, kg/m^2^	29.57 ± 2.31	29.40 ± 3.17	0.697
Smoking, *n* (%)	36 (41)	3 (4)	<0.001
Hypertension, *n* (%)	46 (65)	56 (64)	0.956
Diabetes, *n* (%)	27 (38)	52 (60)	0.007
Dyslipidemia, *n* (%)	32 (45)	48 (55)	0.206
CAD, *n* (%)	30 (42)	49 (56)	0.079
Heart failure, *n* (%)	10 (14)	21 (24)	0.113
Prior myocardial infarction, n (%)	1 (1)	4 (5)	0.380
Stroke/TIA, *n* (%)	3 (4)	6 (7)	0.141
Persistent AF, *n* (%)	28 (39)	39 (45)	0.456
CHA_2_DS_2_VASC score	3 (1, 4)	4 (3, 5)	<0.001

*Values are shown as the mean±SD, median (first to third quartile) or n (%)*.

*AF, atrial fibrillation; BMI, body mass index; CAD, coronary artery disease; TIA, transient ischemic attack*.

**Figure 2:**
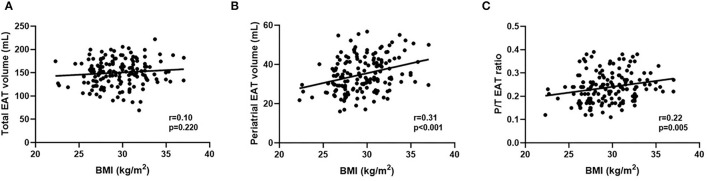
**(A–C)** Correlations between BMI and total EAT volume, periatrial EAT volume and P/T EAT ratio. BMI, body mass index; EAT, epicardial adipose tissue; P/T EAT ratio, proportion of periatrial to total EAT.

Table 2 lists the imaging variables according to sex groups. LVEF, LA dimension, E/A ratio, LAV, and periatrial EAT volume were comparable between sexes. Total EAT volume was significantly lower in female patients than in male patients (140.91 ± 20.31 vs. 161.55 ± 21.65, *P* < 0.001), while female patients had a higher P/T EAT ratio (0.25 ± 0.07 vs. 0.22 ± 0.06, *P* = 0.009) than male patients. BMI was significantly correlated with periatrial EAT volume (*r* = 0.31, *P* < 0.001) and P/T EAT ratio (*r* = 0.22, *P* = 0.005). No significant correlation was found between BMI and total EAT volume (*r* = 0.10, *P* = 0.220; Figure 2). The intraobserver and interobserver reproducibility of total EAT volume and periatrial EAT volume were found to be excellent. The ICC values in the intraobserver analysis were 0.94 (95% confidence interval [CI]: 0.90–0.97) and 0.90 (95% CI: 0.84–0.94) for total EAT volume and periatrial EAT volume, respectively. The ICC values in the interobserver analysis were 0.91 (95% CI: 0.84–0.95) and 0.89 (95% CI: 0.81–0.93) for total EAT volume and periatrial EAT volume, respectively.

**Table 2:** Imaging variables of the total population and stratified according to sex.

	**Male (*n* = 71)**	**Female (*n* = 87)**	**P-value**
LVEF, %	63.14 ± 8.90	63.51 ± 8.62	0.790
LA dimension, mm	38.36 ± 6.15	40.25 ± 6.03	0.053
E/A ratio	1.51 ± 0.82	1.34 ± 0.63	0.144
LAV, mL	100.27 ± 19.94	102.67 ± 21.89	0.473
Total EAT, mL	161.55 ± 21.65	140.91 ± 20.31	<0.001
Periatrial EAT, mL	35.92 ± 9.86	34.28 ± 8.12	0.252
P/T ratio	0.22 ± 0.06	0.25 ± 0.07	0.009

*Values are mean±SD if normally distributed and median (interquartile range) if not normally distributed*.

*LVEF, left ventricular ejection fraction; LA, left atrial; EAT, epicardial adipose tissue. P/T EAT ratio; proportion of periatrial to total EAT*.

**Figure 3:**
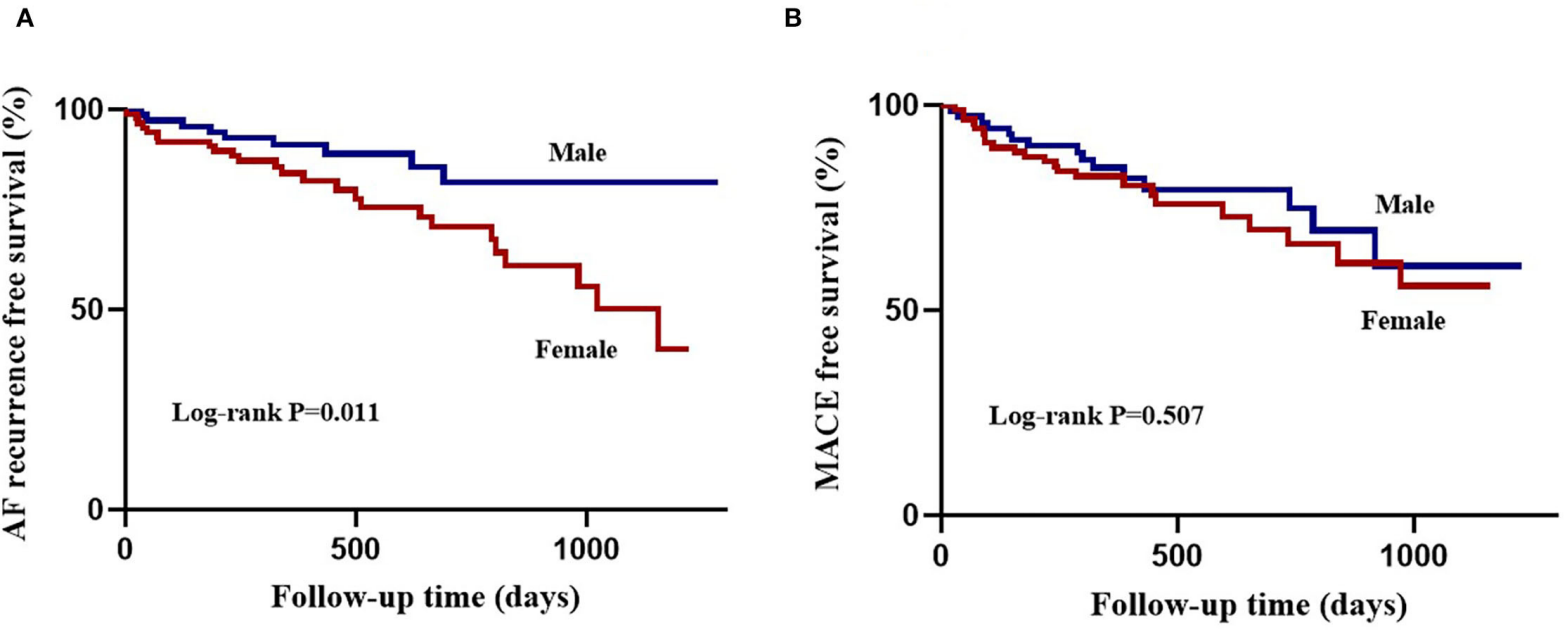
Kaplan-Meier curves of atrial fibrillation outcomes following ablation according to sex. **(A)**, atrial fibrillation recurrence free survival is lower in females compared with males. **(B)**, major adverse cardiovascular events free survival is comparable between sexes.

**Figure 4:**
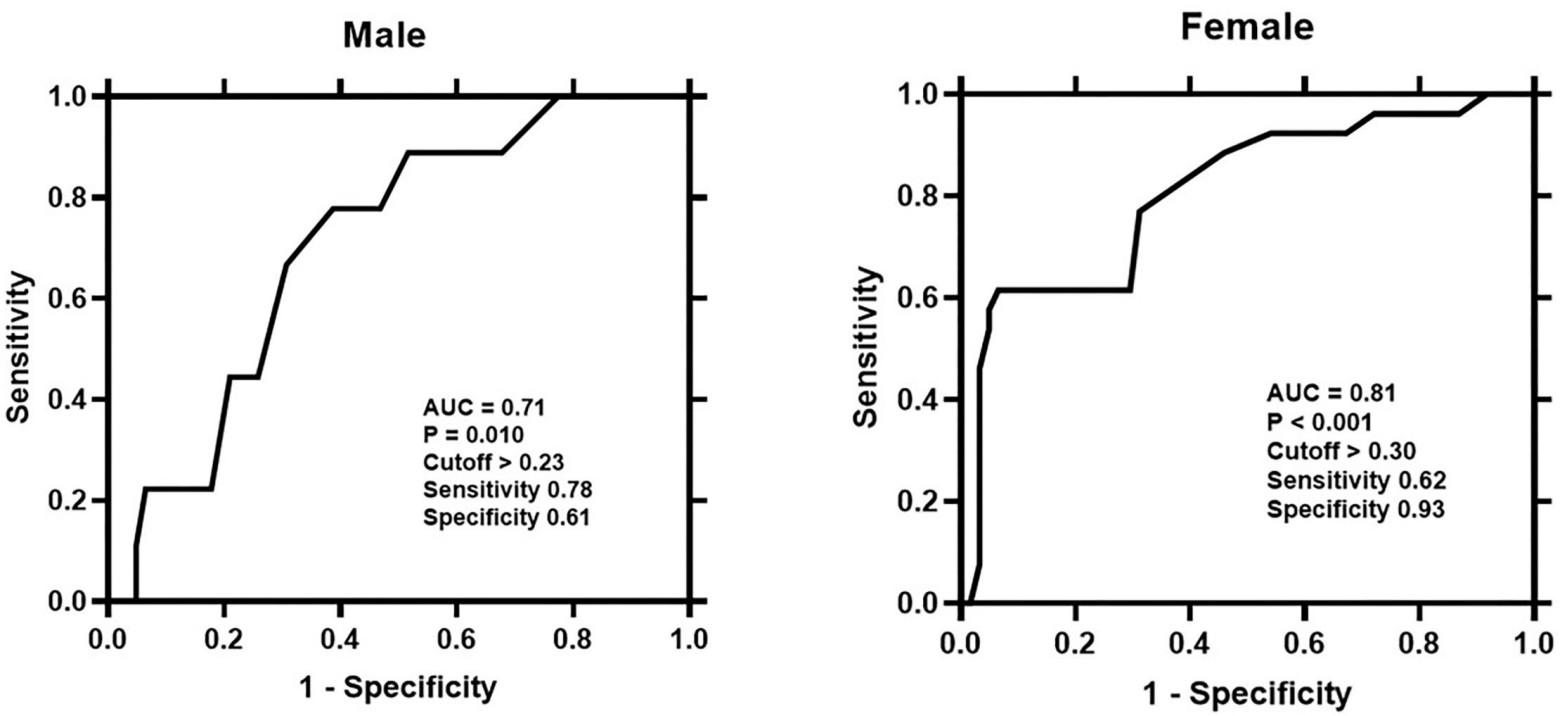
Receiver-operating-characteristic (ROC) curve analysis evaluating the predictive performance of P/T EAT ratio for atrial fibrillation recurrence in the male and female. The cutoff value of P/T EAT ratio for predicting AF recurrence and its sensitivity and specificity were presented. P/T EAT ratio, proportion of periatrial to total EAT.

During the median follow-up period of 444.5 (IQR: 296.3, 804.2) days, female patients had lower arrhythmia-free survival than male patients (26 [30%] vs. 9 [13%], log-rank *P* = 0.011; Figure 3A). Table 3 lists the Cox regression analysis results. In the univariate analysis, the factors that were found to be the predictors of AF recurrence following the catheter ablation were as follows: age (HR: 1.74 [95% CI: 1.14, 2.66], *P* = 0.011), female gender (HR: 2.61 [95% CI: 1.22, 5.60], *P* = 0.014), persistent AF (HR: 2.14 [95% CI: 1.08, 4.24], *P* = 0.030), LA dimension (HR: 1.62 [95% CI: 1.20, 2.20], *P* = 0.002), periatrial EAT volume (HR: 1.72 [95% CI: 1.23, 2.40], *P* = 0.002), and P/T EAT ratio (HR: 1.94 [95% CI: 1.43, 2.64], *P* < 0.001). The result of the multivariate analysis showed that female gender (HR: 1.88 [95% CI: 1.03, 4.15], *P* = 0.032), persistent AF (HR: 2.46 [95% CI: 1.19, 5.05], *P* = 0.015), LA dimension (HR: 1.47 [95% CI: 1.02, 2.13], *P* = 0.041), and P/T EAT ratio (HR: 1.73 [95% CI: 1.12, 2.67], *P* = 0.013) were still the independent predictors of post-ablation recurrence. Subgroup multivariate Cox regression analysis of sex-based showed that P/T EAT ratio remained an independent predictor of AF recurrence in both men (HR: 1.13 [95% CI: 1.01, 1.46], *P* = 0.047) and women (HR: 1.37 [95% CI: 1.11, 1.67], *P* = 0.028) (Supplementary Material 2). ROC analysis demonstrated that the P/T EAT ratio showed good diagnostic performance of AF recurrence in both men (cutoff = 0.23, AUC = 0.71, *P* = 0.010)and women (cutoff = 0.30, AUC = 0.81, *P* < 0.001) (Figure 4).

**Table 3:** Univariable and multivariable cox regression analysis for atrial fibrillation recurrence post-ablation.

	**Univariable analysis**		**Multivariable analysis**	
	**HR (95%CI)**	**P**	**HR (95%CI)**	**P**
Age, y	1.74 (1.14, 2.66)	0.011	1.25 (0.89, 2.25)	0.116
Female	2.61 (1.22, 5.60)	0.014	1.88 (1.03, 4.15)	**0.032**
BMI, kg/m^2^	1.31 (0.94, 1.84)	0.116		
Hypertension	1.66 (0.78, 3.56)	0.189		
Diabetes	1.02 (0.53, 1.99)	0.950		
Dyslipidemia	1.37 (0.69, 2.69)	0.369		
CAD	1.21 (0.62, 2.36)	0.587		
Heart failure	1.49 (0.64, 3.46)	0.355		
Prior myocardial infarction	1.39 (0.33, 5.85)	0.655		
Stroke/TIA	1.75 (0.42, 7.37)	0.447		
Persistent AF	2.14 (1.08, 4.24)	0.030	2.46 (1.19, 5.05)	**0.015**
Anti-arrhythmic drugs	0.98 (0.50, 1.90)	0.942		
LVEF, %	0.92 (0.63, 1.34)	0.664		
LA dimension, mm	1.62 (1.20, 2.20)	0.002	1.47 (1.02, 2.13)	**0.041**
LAV, mL	1.01 (0.99, 1.03)	0.193		
Total EAT, mL	1.39 (0.98, 1.92)	0.052	1.18 (0.77, 1.81)	0.446
Periatrial EAT, mL	1.72 (1.23, 2.40)	0.002	1.18 (0.73, 1.90)	0.502
P/T EAT ratio	1.94 (1.43, 2.64)	<0.001	1.73 (1.12, 2.67)	**0.013**

*BMI, body mass index; CAD, coronary artery disease; TIA, transient ischemic attack; AF, atrial fibrillation; LVEF, left ventricular ejection fraction; LA, left atrial; LAV, left atrial volume; EAT, epicardial adipose tissue. P/T EAT ratio; proportion of periatrial to total EAT. Bold values indicate P < 0.05*.

By the time of the final follow-up, MACE had occurred in 23 (26%) female patients and 15 (21%) male patients (log-rank *P* = 0.507; Figure 3B), including stroke/TIA in 3 (3%) female patients, heart failure in 8 (9%) female patients and 4 (6%) male patients, cardiovascular hospitalization in 17 (20%) female patients and 13 (18%) male patients, and cardiovascular death in 1 (1%) female patient and 1 (1%) male patient. Table 4 shows the major determinants of MACE after ablation. As revealed by the univariable analysis, age (HR: 1.89 [95% CI: 1.27, 2.83], *P* = 0.002), BMI (HR: 1.60 [95% CI: 1.16, 2.20], *P* = 0.004), periatrial EAT volume (HR: 1.43 [95% CI: 1.04, 1.96], *P* = 0.027), and P/T EAT ratio (HR: 1.44 [95% CI: 1.08, 1.91], *P* = 0.012) were significantly correlated with MACE. In the multivariable analysis, age (HR: 1.81 [95% CI: 1.18, 2.77], *P* = 0.007), BMI (HR: 1.44 [95% CI: 1.02, 2.03], *P* = 0.038), and periatrial EAT volume (HR: 1.31 [95% CI: 1.01, 1.91], *P* = 0.046) were found to be independent of MACE post-ablation.

**Table 4:** Univariable and multivariable cox regression analysis for major adverse cardiovascular events post-ablation.

	**Univariable analysis**		**Multivariable analysis**	
	**HR (95%CI)**	**P**	**HR (95%CI)**	**P**
Age, y	1.89 (1.27, 2.83)	0.002	1.81 (1.18, 2.77)	**0.007**
Female	1.25 (0.65, 2.39)	0.508		
BMI, kg/m^2^	1.60 (1.16, 2.20)	0.004	1.44 (1.02, 2.03)	**0.038**
Hypertension	1.52 (0.75, 3.06)	0.245		
Diabetes	1.23 (0.65, 2.33)	0.535		
Dyslipidemia	1.40 (0.73, 2.69)	0.310		
CAD	1.34 (0.70, 2.55)	0.380		
Heart failure	1.09 (0.45, 2.65)	0.846		
Prior myocardial infarction	1.39 (0.33, 5.90)	0.654		
Stroke/TIA	1.12 (0.43, 2.89)	0.823		
Persistent AF	1.03 (0.52, 2.04)	0.939		
Anti-arrhythmic drugs	0.85 (0.45, 1.61)	0.615		
CHA_2_DS_2_VA score	1.39 (0.92, 2.10)	0.114		
LVEF, %	1.05 (0.74, 1.48)	0.790		
LA dimension, mm	1.23 (0.90, 1.69)	0.196		
LAV, mL	1.02 (0.98, 1.07)	0.447		
Total EAT, mL	0.87 (0.63, 1.19)	0.383		
Periatrial EAT, mL	1.43 (1.04, 1.96)	0.027	1.31 (1.01, 1.91)	**0.046**
P/T EAT ratio	1.44 (1.08, 1.91)	0.012	1.01 (0.64, 1.53)	0.971

*AF, atrial fibrillation; BMI, body mass index; CAD, coronary artery disease; TIA, transient ischemic attack; LVEF, left ventricular ejection fraction; LA, left atrial; LAV, left atrial volume; EAT, epicardial adipose tissue. P/T EAT ratio; proportion of periatrial to total EAT. Bold values indicate P < 0.05*.

## Discussion

This study demonstrated that there were significant sex-related differences in the distribution of EAT in patients with AF. Postmenopausal women had a higher P/T EAT ratio while men had a higher total EAT volume. Female patients had a higher incidence of post-ablation AF recurrence but a comparable prevalence of MACE compared with male patients. Female gender and P/T EAT ratio were independent of post-ablation AF recurrence. Sex-based subgroup multivariable analysis showed that the P/T EAT ratio was an independent predictor of AF recurrence in both sexes. While age and periatrial EAT volume were independent predictors of MACE.

### EAT and AF

It is generally known that the risk of AF is higher in men compared with women, even after adjusting for age (22). Reasons for the differences may comprise sex-specific atrial electrophysiologic properties, atrial remodeling, and mechanisms of atrial fibrosis (23). Obesity-related AF has been gradually increased, and obesity has been considered a crucial and modifiable risk factor for AF development (11, 24, 25). However, obesity can be an attribute of general adiposity with variable degrees of cardiovascular and metabolic presentations. EAT, the more specific fat depot, has aroused rising attention for its close anatomic proximity to coronary arteries and crosstalk with cardiomyocytes. Some evidence has demonstrated that EAT can play a certain role in the pathogenesis and progression of AF, and the relationship was not dependent on total adiposity and LA enlargement (11, 14, 26). A Framingham Heart Study of 2,317 patients discovered that a higher total EAT volume was significantly associated with 40% higher hazards of AF independent of traditional risk factors (13). In addition to total EAT, existing studies further suggest that specific periatrial EAT is more significantly correlated with AF, and it may directly facilitate structural and electric remodeling (11, 27). Van Rosendael et al. suggested that for every gram increase in posterior LA adipose tissue mass, the risk of AF increased by 32% (28).

While the mechanisms correlating EAT with AF were unclear, some potential hypotheses were proposed in accordance with existing prior studies. One of the vital extrapolations may be the role played by EAT in the AF electrophysiological substrate. Direct infiltration of lipocytes within the atrial myocardium could accelerate side-to-side cells' connection loss and induce remodeling of the atrial substrate. Moreover, adipocyte infiltration interpenetrating between myocytes was implicated in decreased voltage and increased voltage heterogeneity within the posterior left atrium, thus jointly leading to subsequent conductive defects (conduction deceleration or heterogeneity) (29–31). A study using a distinctive 3D merge process and dominant frequency LA map determined that fat locations corresponded to high dominant frequency. EAT co-localizes with high dominant frequency areas, revealing that it could be most likely to harbor high-frequency regions, creating beneficial circumstances for the perpetuation of AF (32, 33). For another potential mechanism, EAT was found as the anatomical location of the cardiac intrinsic autonomic nervous system (e.g., ganglionated plexi (GP) and interconnecting nerves) located near the pulmonary veins (34, 35). The ganglia were the key determinants responsible for triggering and perpetuating AF (36). GP activation contributed to the short action potential duration and increased calcium release, thus evoking triggered firing due to a delayed post-depolarization of the atrial/pulmonary vein, as demonstrated by the high dominant frequency sites (35). Another additional mechanism was that EAT, as an endocrine organ, could produce variable pro-inflammatory cytokines, pro-fibrotic factors, reactive oxygen species, and adipocytokines through paracrine or vasocrine pathways, which exerted deleterious local effects on the atrium (e.g., functional disorganization) and further structurally remodeled and produced arrhythmogenic substrates (35). Matrix metalloproteinases, a vital modulator of extracellular matrix turnover that contributes to atrial fibrosis, were upregulated in the AF period, and their secretion increased within EAT (37, 38). An existing study reported that female patients with AF had more advanced atrial remodeling compared with male patients (39).

### EAT and AF Recurrence

Some reports found sex differences in the AF recurrence (7, 8, 39). Consistent with the above results, this study demonstrated that women experienced a higher incidence of post-ablation AF recurrence. However, the mechanisms of the sex-related differences remain unclear. It has been assumed that periatrial EAT is more significantly correlated with AF recurrence after catheter ablation (20, 40, 41). EI Mahdiui et al. quantified posterior LA EAT attenuation and explored its relationship with AF recurrence following catheter ablation (41). They reported that patients suffering from higher posterior LA EAT attenuation had a significantly more frequent recurrence rate, and the fat attenuation could serve as a promising predictor of AF recurrence (41). Existing studies evaluated both periatrial and periventricular EAT and found both the fat in the two regions were correlated with AF recurrence, but the closer relationship existed in periatrial EAT (31, 40, 42). As revealed by the above results, EAT promoted AF recurrence to varying extents in different myocardial sites, and the evaluation of EAT around the left atrium might be a preferable methodological evaluation (41). Our study showed that total EAT volume was significantly higher, whereas the P/T EAT ratio was lower in male patients than in female patients. In general, body fat distribution has been different between the sexes: visceral fat obesity predominates in men, while subcutaneous fat obesity predominates in women (43). Existing studies reported that although men had significantly larger pericardial fat volumes than women, the adipose was correlated with more adverse risk factors in women (44). Kim et al. reported that women had more periatrial adiposity, which probably adversely affected LA voltage and transport function (45). The bioactivity of periatrial EAT may be affected by the degree of periatrial adiposity, and metabolically abnormal periatrial EAT further affects the atrium, leading to atrial remodeling and dysfunction (45). Besides, female patients and the P/T EAT ratio instead of periatrial or total EAT were independently correlated with AF recurrence. The above findings reveal that a relatively abundant P/T EAT ratio in women may affect AF recurrence on the basis of several potential mechanisms: Catheter ablation targeted regions for substrate modifications overlap with the majority of LA EAT and adipose tissue had a lower electrical conduction than atrial myocardium, and the increased amount of periatrial EAT may directly decrease the opportunity for successful procedure (35, 46). Furthermore, atrial fibrosis as a result of a fat-induced local inflammatory course may facilitate the development of intra-atrial reentry circuits, thus decreasing the success of catheter ablation (47, 48).

### EAT and MACE

Some recent studies have demonstrated EAT as a risk factor for future adverse cardiovascular events in CAD, diabetes, and even asymptomatic populations (14, 49, 50). Eisenberg et al. assessed the prognostic value of EAT volume and attenuation with the use of deep learning-based algorithms quantification from non-contrast cardiac computed tomography in 2,068 asymptomatic individuals with 14 ± 3 years of follow-up (49). They reported that EAT volume and attenuation could be employed to predict MACE independent of traditional risk factors and coronary calcified score (49). Christensen et al. revealed that a high amount of EAT were correlated with incident cardiovascular disease and mortality in patients with type 1 diabetes (T2DM) after they were adjusted for cardiovascular risk factors (50). However, no research has been conducted on the effect of periatrial EAT on MACE in patients with AF following the catheter ablation. Accordingly, the present research extended previous findings to AF patients and explored the correlation between periatrial EAT volume and the incidence of MACE, determining whether there existed sex-related differences. This study revealed that women were not an independent risk factor for MACE in AF patients with post-ablation. The results of a large prospective study suggested that although female patients had a higher incidence of thromboembolism than male patients, they did not emerge as an independent risk factor for stroke and systemic embolism (51). Furthermore, this study also found that periatrial EAT volume was independently correlated with MACE, suggesting that therapeutic targeting of periatrial EAT for MACE prevention in patients with AF post-ablation is promising. A recent review also supports the concept that EAT is a central pathogenetic mechanism and therapeutic target for AF (52).

### Study Limitations

This study had several limitations. First, there was a limited sample size with a single center design and retrospective data evaluation, which probably limited evaluation of causality. Accordingly, the above findings should be confirmed in a larger and prospective cohort. Second, the follow-up period was relatively short to determine clinical events, which may affect our results. Third, the results of this study might not be extrapolated to other ethnical populations, since the demographics in this study were dominated by Chinese.

## Conclusion

Through the comparison of the sex-based differences in the EAT distribution and AF outcomes post-ablation, it was reported that postmenopausal women had a higher P/T EAT ratio and a higher incidence of AF recurrence than men, but both sexes had similar rates of MACE. In addition, female gender and P/T EAT ratio were found to be independent predictors of AF recurrence, and P/T EAT ratio is still independently associated with AF recurrence in both sexes. While age and periatrial EAT volume were independently correlated with MACE, which suggested that the difference in the distribution of EAT might have an effect on MACE, and the regional distribution of EAT depending on sex may have an impact on AF recurrence.

## Data Availability Statement

The raw data supporting the conclusions of this article will be made available by the authors, without undue reservation.

## Author Contributions

JZ designed study, wrote the study, reviewed the study, gave input to improve the study, and read and approved the final manuscript. KZ collected data, measured imaging parameters, performed research, and wrote the study. BZ reviewed and analyzed data and performed the data interpretation. ZX measured imaging parameters. WL reviewed data. All authors contributed to the article and approved the submitted version.

## Conflict of Interest

The authors declare that the research was conducted in the absence of any commercial or financial relationships that could be construed as a potential conflict of interest.

## Publisher's Note

All claims expressed in this article are solely those of the authors and do not necessarily represent those of their affiliated organizations, or those of the publisher, the editors and the reviewers. Any product that may be evaluated in this article, or claim that may be made by its manufacturer, is not guaranteed or endorsed by the publisher.
