# Supplementary material for: Sex Differences in Epicardial Adipose Tissue: Association With Atrial Fibrillation Ablation Outcomes
Source: Front Cardiovasc Med. 2022 Jun 13;9:905351. doi: 10.3389/fcvm.2022.905351 (PMC9234200; doi:10.3389/fcvm.2022.905351)
Supplement: Supplementary file 1 [file Data_Sheet_1.docx]

**Supplement 1 baseline characteristics of the study populations**

|  | Male (n=71) | Female (n=87) | P-value |
| --- | --- | --- | --- |
| Medication, n (%) |  |  |  |
| Warfarin | 29 (41) | 35 (40) | 0.938 |
| Aspirin | 3 (4) | 4 (5) | 0.910 |
| Novel oral anticoagulants | 38 (54) | 49 (56) | 0.725 |
| Anti-arrhythmic drugs | 42 (59) | 41 (40) | 0.132 |
| Statins | 12 (17) | 20 (23) | 0.344 |
| ACE inhibitor/ARB | 11 (15) | 17 (20) | 0.508 |
| β-Blocker | 17 (24) | 28 (32) | 0.254 |
| Calcium antagonist | 11 (15) | 17 (20) | 0.508 |
| Diuretics | 8 (11) | 15 (17) | 0.290 |

Values are shown as n (%).

ACEI, angiotensin converting enzyme inhibitor; ARB, angiotensin II receptor blocker.

**Supplement 2 Univariable and multivariable cox regression analysis for atrial fibrillation recurrence post-ablation in male and female**

|  | Male |  |  |  |  |  | Female |  |  |  |  |
| --- | --- | --- | --- | --- | --- | --- | --- | --- | --- | --- | --- |
|  | Univariable analysis |  |  | Multivariable analysis |  |  | Univariable analysis |  |  | Multivariable analysis |  |
|  | HR (95%CI) | P |  | HR (95%CI) | P |  | HR (95%CI) | P |  | HR (95%CI) | P |
| Age, y | 1.07 (0.99, 1.15) | 0.093 |  | 1.04 (0.97, 1.12) | 0.230 |  | 1.06 (1.00, 1.12) | 0.041 |  | 1.07 (1.02, 1.13) | 0.013 |
| BMI, kg/m^2^ | 1.39 (1.12, 1.73) | 0.003 |  | 1.23 (1.02, 1.54) | 0.035 |  | 1.01 (0.89, 1.15) | 0.887 |  |  |  |
| Hypertension | 1.94 (0.40, 3.36) | 0.408 |  |  |  |  | 2.08 (0.83, 4.21) | 0.119 |  |  |  |
| Diabetes | 1.21 (0.30, 4.83) | 0.791 |  |  |  |  | 1.41 (0.63, 3.15) | 0.398 |  |  |  |
| Dyslipidemia | 1.53 (0.94, 1.83) | 0.059 |  | 3.43 (0.62, 6.10) | 0.159 |  | 1.41 (0.64, 3.11) | 0.397 |  |  |  |
| CAD | 1.83 (0.55, 3.05) | 0.325 |  |  |  |  | 1.98 (0.89, 4.41) | 0.093 |  |  |  |
| Heart failure | 1.07 (0.77, 2.30) | 0.113 |  |  |  |  | 1.01 (0.34, 3.01) | 0.990 |  |  |  |
| Persistent AF | 1.61 (0.42, 3.16) | 0.486 |  |  |  |  | 1.65 (1.15, 2.09) | 0.022 |  | 1.74 (1.01, 2.46) | 0.049 |
| Anti-arrhythmic drugs | 0.37 (0.08, 1.81) | 0.221 |  |  |  |  | 0.92 (0.42, 2.01) | 0.827 |  |  |  |
| LVEF, % | 1.01 (0.93, 1.09) | 0.889 |  |  |  |  | 1.02 (0.97, 1.08) | 0.446 |  |  |  |
| LA dimension, mm | 1.12 (1.01, 1.24) | 0.040 |  | 1.09 (0.95, 1.25) | 0.216 |  | 1.08 (1.02, 1.14) | 0.012 |  | 1.06 (0.97, 1.16) | 0.183 |
| LAV, mL | 1.01 (0.98, 1.04) | 0.701 |  |  |  |  | 1.02 (1.00, 1.04) | 0.030 |  | 1.02 (0.99, 1.05) | 0.068 |
| Total EAT, mL | 1.02 (0.99, 1.04) | 0.278 |  |  |  |  | 0.99 (0.97, 1.00) | 0.064 |  | 1.05 (0.95, 1.15) | 0.362 |
| Periatrial EAT, mL | 1.08 (1.00, 1.15) | 0.041 |  | 1.01 (0.90, 1.23) | 0.297 |  | 1.07 (1.02, 1.13) | 0.005 |  | 1.03 (0.62, 1.69) | 0.356 |
| P/T EAT ratio | 1.24 (1.02, 2.26) | 0.039 |  | 1.13 (1.01, 1.46) | 0.047 |  | 1.65 (1.31, 2.03) | 0.001 |  | 1.37 (1.11, 1.67) | 0.028 |

BMI, body mass index; CAD, coronary artery disease; AF, atrial fibrillation; LVEF, left ventricular ejection fraction; LA, left atrial; LAV, left atrial volume; EAT, epicardial adipose tissue. P/T EAT ratio; proportion of periatrial to total EAT.
